# PReS-FINAL-2050: Determination of the concentration of methotrexate in the blood serum of patients with JIA

**DOI:** 10.1186/1546-0096-11-S2-P63

**Published:** 2013-12-05

**Authors:** F Rokhlina, G Novik

**Affiliations:** 1 SPbGPMU, St. Petersburg, Russian Federation

## Introduction

Juvenile idiopathic arthritis (JIA) is the most common rheumatic disease among children. Among patients with JIA often meets resistance to the therapy of various drugs, including nonsteroidal anti-inflammatory drugs (NSAIDs), intraarticular injections and physiotherapy. For the treatment of JIA is used several groups of drugs: NSAIDs, corticosteroids (CS), immunosuppressive drugs and biologically active substances obtained genno-ingenerian. Application NSAIDs and GS have anaesthetic and anti-inflammatory effects, but do not contribute to the prevention of destruction of the joints and disability of patients. The ≪gold standard≫ in the treatment of JIA can be considered methotrexate (MTX) - cytostatic drug groups of antimetabolites, antagonists folic acid. MTX has expressed immunosupression action, even at relatively low doses, with no noticeable hematologic toxicity. Despite this, when taking methotrexate recommended clinical and laboratory monitoring is needed to prevent side effects. According to various authors, about 70% of patients receiving MTX consists in the remission of the disease.

## Objectives

The aim of our study is to define the efficient MTX concentration in the blood serum of patients with JIA.

## Methods

All children included in the study received basic therapy with methotrexate dose of 15 mg/m2 over 3 months and more. All the children were conducted routine methods of research, objective examination and determined the MTX concentration in the serum.

## Results

We examined 103 child patients with JIA, among which - 65 (63,11%) girls and 38 (36,89%) boys with various forms of juvenile idiopathic arthritis. Children with polyarthritis - 46 (44,66%), with olygoarthritis JIA - 25 children (24,27%), systemic type of arthritis diagnosed with 14 children (13,59%), enthesitassociated arthritis met in 18 patients (17,48%).

35 patients (34%) to the treatment with MTX added biologically active therapy, 11 patients (10,68%) receive in addition to the MTX therapy sulfasalazine, 24 child (23,3%) - cyclosporine A, 37 children (35,92%) being prednisone or methylprednisone (in tablets or in the form of a pulse therapy). Noteworthy is that 46 (44,66%) patients have the positive effects of MTX monotherapy, does not require the appointment of additional drugs.

## Conclusion

According to the information we received data MTX concentration in the serum was in the range of 0.76 mmol/lIA to 3,96 mmol/L. Significant differences depending on the activity of the disease, type of arthritis, treatment duration MTX were received. Thus the definition of MTX concentration in the blood serum of patients with JIA not let to forecast the efficiency of the therapy.

MTX has a good safety profile. To reduce the risk of side effects, appoint folic acid at a dose of 1-5 mg) in the days when MTX is not used, because while receiving folic acid may reduce the effectiveness of MTX. An important feature of the use of this drug is to use 1 time a week.

The use of cytostatic therapy (MTX) is the drug of choice in all countries for the treatment of various forms of JIA and is the gold standard.

## Disclosure of interest

None declared.

